# Biomarkers for Osteoarthritis Diseases

**DOI:** 10.3390/life12111799

**Published:** 2022-11-07

**Authors:** Jacob A. Braaten, Mark T. Banovetz, Nicholas N. DePhillipo, Filippo Familiari, Raffaella Russo, Nicholas I. Kennedy, Robert F. LaPrade

**Affiliations:** 1 University of Minnesota Medical School, Minneapolis, MN 55455, USA; 2 Department of Orthopaedic Surgery, University of Pennsylvania, Philadelphia, PA 19104, USA; 3 Department of Orthopaedic and Trauma Surgery, Magna Graecia University, 88100 Catanzaro, Italy; 4 Department of Medical and Surgical Sciences, Magna Graecia University, 88100 Catanzaro, Italy; 5 Twin Cities Orthopedics, 4010 W 65th St, Edina 55435, MN, USA

**Keywords:** osteoarthritis, biomarker, inflammation

## Abstract

Growing evidence has revealed the pivotal role of inflammatory biomarkers in the pathogenesis of osteoarthritis. There is significant interest in the prognostic value of select biomarkers, given the potential for early identification and treatment of patients at risk of osteoarthritis prior to the development of irreversible clinical disease. Clinical trials of novel therapeutics that disrupt the inflammatory pathways of osteoarthritis are also ongoing. The purpose of this review is to summarize the current literature on key biomarkers within the context of osteoarthritis pathogenesis, clinical symptom development, and treatment capabilities. Multiple recent studies have established biomarkers that signal the existence of osteoarthritis pathology and the development of clinical symptomology. However, prior to implementation in clinical practice, additional research is required to precisely define the prognostic value for numerous biomarkers and standardize their measurement. Biomarker-driven investigations represent a promising avenue for the early diagnosis and treatment of osteoarthritis.

## 1. Introduction

Osteoarthritis (OA) describes a specific subset of the pathological processes affecting the joint in degenerative joint disease (DJD) [1,2]. Whereas DJD characterizes the degeneration of all tissues around the synovial joint, including the metaphyseal bone, ligaments, capsule, and articular cartilage, OA involves the chronic, progressive degradation of articular cartilage [1,2]. Osteoarthritis is remarkably prevalent, representing one of the most common chronic disease processes in the Western world [1,3,4]. With an estimated prevalence that is well into the tens of millions in the United States alone, the incidence continues to grow as the population continues to age and suffers increasing rates of obesity, as OA represents a disease process with a distinct predilection for advanced age and high body mass index (BMI) [1,3]. Despite the prevalence of this disease, the treatments for OA remain mostly palliative.

Traditionally, OA was a disease that was often, if not exclusively, described at the gross level. The rising focus on biochemical markers for understanding the pathologic basis of disease in the late 20th and early 21st centuries is a trend that has not excluded OA. Generally speaking, the study of OA at the microscopic and molecular level has elucidated principles of the disease that could not otherwise be ascertained by solely continuing to study the disease at the gross level. After all, it is through the study of relevant biomarkers and their role in the pathophysiologic process leading to OA that the observation has been made that the process of OA begins long before both clinical symptoms required for diagnosis and gross morphological changes become apparent to the clinician [2]. 

A systematic review by Boffa et al. defines a “biomarker” as a characteristic that is objectively measured and evaluated as an indicator of normal biological processes, pathogenic processes, or pharmacologic responses to therapeutic intervention, and it can be represented by radiographic, histologic, physiologic, or molecular characteristics [2]. Kumavat et al. describe the process of discovering, identifying, and trialing new biomarkers as analogous to the process by which drugs and their targets are identified and studied (i.e., in a very particular stagewise manner) [3].

It has been estimated that worldwide approximately 300 million people have radiographic evidence of OA, whether it be occult or symptomatic [1]. Thus, the importance of biomarkers for describing the disease process before the onset of symptoms and/or during the exploration of novel pharmacotherapeutics cannot be understated. 

The purpose of this review was to report on the currently relevant biomarkers of OA disease. This review first provides an overview of the pathogenic process leading to OA at the cellular and molecular levels. In doing so, it will introduce the key cellular and molecular players in this process such that an intuitive association can be made between the currently relevant biomarkers for this process and the pathologic process which makes them relevant. This review also discusses future directions for the use of biomarkers in the diagnosis and treatment of OA, as well as their currently understood limitations.

## 2. Pathogenesis of Osteoarthritis

Initially characterized as the progressive loss of articular cartilage, the pathogenesis of OA is now known to involve multiple constituent tissues of the synovial joint, including the synovium, subchondral and metaphyseal bone, and the stabilizing periarticular ligaments and muscles of the joint capsule [1,5,6]. The development of OA occurs due to an alteration of joint tissue repair and breakdown homeostasis. With the progression of the disease, the balance shifts towards joint tissue degradation and results in the development of synovial inflammation, subchondral bone remodeling, osteophyte formation, and diminished strength of periarticular muscles. Over time, these structural changes produce the clinical signs of OA, including joint pain, stiffness, crepitus, effusions, and decreased knee motion [6]. Early treatment of OA is required to prevent the development of joint failure [1].

The pathogenesis of OA has been reported as having three overlapping stages [7]. The first stage of OA pathogenesis involves molecular-level damage to the extracellular matrix (ECM) of the articular cartilage surface. Multiple risk factors have been reported to contribute to ECM disruption, including age, inactivity, and chronic supraphysiologic biomechanical forces on the knee joint [8,9]. In addition, environmental, genetic, metabolic, and biochemical processes have also been associated with more severe OA outcomes [8,9,10]. The degeneration of the ECM results in the development of articular cartilage clefts and fibrillation that, with repeated insults to the articular cartilage, deepens to reach the subchondral bone. This subchondral microdamage contributes to alterations of joint shape and load transmission that propagates additional cartilage damage [1,7]. 

Over time, the ECM cartilage tears and releases free fragments composed of fibronectin and type II collagen into the synovium [11]. The ECM fragments are broken down by newly-activated synovial cells that release a host of pro-inflammatory and catabolic biochemical markers and facilitate the production of matrix metalloproteases (MMPs) by chondrocytes to initiate a vicious cycle of subsequent articular cartilage breakdown [12,13]. The synovial biochemical markers also promote the inflammatory cell infiltration of the synovium by lymphocytes and macrophages and increase the concentration of additional inflammatory markers, such as C-reactive proteins and interleukin-1 (IL-1) [7,14]. 

The second stage of OA development involves the chondrocyte response to articular and subchondral tissue damage. Initially, chondrocytes in OA respond with up-regulated tissue repair activity, producing type II collagen and anabolic cytokines such as transforming growth factor-beta (TGF-β), insulin-like growth factor-I (IFG-I), bone morphogenic proteins (BMPs) and fibroblast growth factors that restore ECM homeostasis [1,15]. Furthermore, chondrocytes produce alkaline phosphatase, annexins, and type X collagen, representing biochemical markers that facilitate endochondral ossification [16]. This maladaptive process replaces cartilage with bone tissue, worsening existing cartilage injury through progressive calcification and thickening the subchondral bone to form the characteristic subchondral sclerosis of progressive OA [17,18,19]. 

The third stage of OA development is characterized by the failure of chondrocytes to meet the biosynthetic demand for tissue repair in response to degrative catabolic activity. Due to factors such as age and genetics, the chondrocytes are unable to keep up with the progressive damage to articular cartilage and subchondral bone [1]. At this stage, a corresponding decrease in biochemical inhibitors of catabolic cytokines and MMPs, such as tissue inhibitors of metalloproteinases (TIMPs)-1, is observed [20]. Continued degeneration of the tissue results in alteration of the biochemical environment and reduced cellularity of tissue via increased chondrocyte cell death, a hallmark of severe OA [1,21,22].

## 3. Clinical Relevance of Biochemical Markers for Osteoarthritis

The role of biomarkers in OA diagnosis, disease progression, and treatment has gained attention in recent years. Multiple studies have investigated the predictive value of biomarkers to determine clinical disease severity, while other researchers have focused on the development of new OA therapies that target biomarker production and regulation [23,24]. The latest evidence suggests that there exist pre-symptomatic biomarkers for OA, a disease often accompanied by a prolonged asymptomatic clinical period with the concomitant progression of tissue destruction. The current diagnosis of OA is based on clinical symptoms of joint pain, stiffness, and loss of mobility, coupled with radiographic evidence of the disease, such as joint space narrowing, subchondral sclerosis, cyst formation, and osteophyte development, which often appear at a late, irreversible stage [1,2,7]. As such, the treatment of OA remains largely palliative. 

If accurately detected, the early identification of OA biomarkers may allow for more rapid treatment compared to currently established means and may delay the sequelae of cartilage and bone damage that results in clinical disability [4,23,24]. Furthermore, individualized biomarker evaluation as a form of personalized medicine holds promise to predict and/or monitor the therapeutic response to conventional OA treatment with therapies such as nonsteroidal anti-inflammatory drugs (NSAIDs), corticosteroids, and hyaluronic acid [2]. Therefore, biomarker-driven investigations have exponentially increased over the past decade, given the potential for biomarkers to serve as both an OA screening tool and a cornerstone of therapy. 

In 2012, the Foundation for the National Institutes of Health (FNIH) and the Osteoarthritis Research Society International (OARSI) developed a consortium with a two-fold purpose. The first was to standardize the scientific steps of biomarker investigations related to the development of therapeutics for OA and establish factors such as age, gender, physical activity, and circadian variation that may affect baseline measurements [25,26]. The second was to identify highly validated biomarkers that were predictive of OA clinical outcomes [24]. As a result of the consortium, several trials were initiated that have investigated the role of specific biomarkers in the pathogenesis of OA, the value of biomarkers as a harbinger of clinical outcomes, and the efficacy of new therapies that target pathologic OA biomarkers [24,27,28,29].

Originating from the work of the FNIH and OARSI, the process of biomarker validation requires precisely defining the biomarker based on sensitivity, specificity, false positive probability, and false negative probability. The specific bioanalytical method of biomarker laboratory assessment must also be considered [2,3]. In subsequent years, additional validation criteria have been proposed. For example, the outcome measures for the rheumatoid arthritis clinical trials (OMERACT) group added truth and feasibility as criteria. In doing so, the authors suggested that a valid biomarker should provide an unbiased measure of the intended target (truth) and that a feasible biomarker should also be measured easily and pragmatically without significant burdens of time, money, or interpretation (feasibility) [3].

## 4. Biochemical Markers of Cartilage Formation and Degradation in Osteoarthritis

The synthesis, and eventual destruction, of type-II collagen, represents the foundational pathophysiology of OA. Synthesized from procollagen in chondrocytes, mature collagen production occurs following the cleavage of procollagen carboxy-terminal (PIICP) and amino-terminal (PIIANP) propeptides. These peptides (Table 1) represent indicators of type-II collagen synthesis and have been reported to be inversely correlated with cartilage destruction in patients with OA and hold promise as a means of early detection of OA [1,30,31]. 

### 4.1. Cartilage Oligomeric Matrix Protein (COMP)

Previous studies have identified numerous biomarkers that indicate cartilage damage and may offer prognostication of OA severity and response to treatment [32]. Cartilage Oligomeric Matrix Protein (COMP) is synthesized by chondrocytes, synovial cells, osteoblasts, and tendon fibroblasts and has been established as a known marker of cartilage demolition that may correlate with the severity of disease in OA [33,34]. However, given the production of COMP from multiple tissues, the elevation remains a nonspecific biomarker that may limit the diagnostic utility for OA [33].

### 4.2. Col2-1 and Col2-1 NO2

Col2-1 and its nitrated form Col2-1 NO2 are peptides within the triple helix structure of the type-II collagen network that have been reported to correlate positively with increased disability in OA patients due to oxidative degradation of Col2-1 following articular cartilage damage. Urinary levels of Col2-1 and Col2-1 No2 may therefore indicate the clinical progression of OA [1]. 

### 4.3. CTX-II

C-terminal cross-linked telopeptide of type II collagen (CTX-II) is a degradation product of type-II collagen and has been identified as a key indicator of articular cartilage damage and joint erosion. After collagen breakdown by collagenase, CTX-II represents a C-terminal telopeptide of epitopes that is released from the collagen triple helix and has been identified as a specific indicator for OA [1]. A study of patients with early-stage OA reported that CTX-I and CTX-II levels in urine might also establish the presence of focal cartilage lesions that precede OA development. Multiple studies in patients with knee and hip OA have found urine CTX-II levels to be a diagnostic marker of current joint damage and increase the risk of rapid OA progression [35,36,37]. 

### 4.4. CXCL12 and CXCR2

CXCL12 is a cytokine that mobilizes mesenchymal stem cells to facilitate soft tissue repair and bone remodeling via binding to the CXCR4 receptor. The role of CXCL12 is complex and has been reported to facilitate both chondrocyte homeostasis after collagen damage and the proinflammatory response during OA [38,39]. Xu et al. reported that elevated synovial fluid CXCL12 levels were correlated with poor radiographic outcomes in OA patients [38]. In addition, CXCR2 is a chemokine that plays a central role in the preservation of chondrocyte health. In a mouse model, a study reported that blockade of CXCR2 led to increased OA severity compared to mice with normal CXCR2 signaling [40].

## 5. Biochemical Markers of Synovial Inflammation

Synovial inflammation in OA occurs due to the release of hyaline-based cartilage products into the joint space, a process that activates synovial cells. In turn, synovial cells produce catabolic, pro-inflammatory biomarkers and proteolytic enzymes, such as MMPs, CXCL13, and CXC, that intensify articular cartilage breakdown and recruit lymphocytes and macrophages to the joint space [41,42]. Recent studies have reported multiple synovial biomarkers to be predictive of OA clinical development and prognosis [43,44,45]. 

### MMPs and TIMP-1

Matrix metalloproteinases (MMPs) regulate enzymatic cartilage ECM degradation and have been implicated in the induction of OA [46,47,48]. Heightened expression of MMP-13, a specific MMP enzyme, has been observed in the cartilage of OA patients compared to normal adult cartilage [48]. Furthermore, MMP-13 has been associated with the rapid progression of OA due to the significant proteolytic capacity of type-II cartilage [48]. MMP-3 levels have also been positively correlated with joint space narrowing in OA patients and may suggest increased clinical severity of the disease [49].

Therapeutics targeting MMPs in OA have shown promise for slowing the extent of cartilage erosion and subsequent clinical deterioration. Tissue inhibitor of metalloproteinases (TIMPs-1) suppresses MMP activity and may become an option for preventative treatment of OA. However, data is limited at present, and additional studies are warranted [43,45].

## 6. Biochemical Markers of Joint Injury and Inflammation

Growing evidence has shed light on the role of inflammation in OA pathogenesis and clinical development. Previously thought to be secondary to chronic biomechanical “wear and tear”, OA inflammatory biomarkers (Table 2) are now known to be integral to cartilage degradation and joint remodeling mechanisms [39]. As a result, experimental therapies which target inflammatory pathways are currently of considerable interest, given the potential for novel, anti-inflammatory-based treatments to decrease the progression and onset of clinical symptoms in OA.

### 6.1. CCL2

Multiple cytokines within the chemokine (CC) family have been implicated in the development of OA. Chemokine ligand 2 (CCL2) is a member of the CC family that recruits T-lymphocytes and natural killer (NK) cells to the joint space during joint injury. CCL2 has also been found to increase the expression of MMP-3 in OA, exacerbating the loss of articular cartilage. Past studies have found increased synovial CCL2 levels in patients after ankle and knee injury, indicating the inflammatory function of CCL2 in joint damage [50,51,52]. A study of 18 patients reported that increased levels of CCL2 in the knee synovium were associated with evidence of radiographic OA development [52]. CCL2 may also be associated with the onset of pain in knee OA through stimulation of dorsal root ganglia nociceptive pathways [53]. Although these findings indicate that CCL2 contributes to inflammation, pain, and cartilage degradation in OA, additional research is required to establish the specific role of CCL2 as a diagnostic and predictive marker. 

### 6.2. CRP

Although OA lacks the systemic inflammatory response of rheumatoid arthritis, past studies have identified the pathogenic role of pro-inflammatory acute phase reactants in OA [54]. C reactive protein (CRP) is the most commonly studied acute phase reactant and has been associated with the onset of OA clinical symptoms, although there is varying evidence in the literature [55]. A systematic review by Jin et al. reported a “modest elevation” of high sensitive CRP (hs-CRP) in patients with OA and found a significant relationship between elevated hs-CRP levels and the clinical development of physical limitations and pain [55]. Notably, the authors did not find a significant relationship in the literature between hs-CRP levels and evidence of radiographic OA progression, such as joint space narrowing [55]. Overall, it is likely that CRP facilitates a low-grade inflammatory state that exacerbates clinical symptoms without significant contribution to macroscopic degenerative joint changes. 

### 6.3. Ghrelin

The anti-inflammatory role of the neuropeptide ghrelin in OA pathophysiology has recently attracted attention in the literature. The primary arthritic function of ghrelin is to facilitate chondrocyte differentiation and metabolism [56,57]. Ghrelin has been reported to inhibit the synthesis and function of pro-inflammatory cytokines such as IL-1β and TNF-α through suppression of the NF-κβ pathway [58]. Studies have reported elevated ghrelin levels in multiple inflammatory diseases, such as ankylosing spondylitis, pancreatitis, sepsis, and arthritis, that significantly correlate with disease progression [57,59,60]. 

At present, the exact mechanism of ghrelin in OA development is unclear. It has been proposed that observed increases in ghrelin in OA may be due to the anti-inflammatory role of ghrelin in attenuating the systemic inflammatory response [61]. A recent study by Wu et al. reported significantly increased serum ghrelin levels in patients with clinical symptoms of knee OA, characterized by WOMAC scores that assess knee stiffness, pain, and impairment [57]. Ghrelin may also exhibit anti-nociceptive properties in OA, although the precise effect needs to be established by additional studies [57]. Conversely, a study reported that increased ghrelin levels were inversely correlated with inflammatory marker concentrations and biomarkers of cartilage degradation in patients with knee post-traumatic OA, such as TNF-α, IL-6, COMP, and CTX-II [62]. Despite the unclear mechanism, elevated synovial ghrelin levels have been associated with improved clinical outcomes in OA and may represent a candidate for the evaluation of injury progression [62]. 

### 6.4. Leptin

Leptin predominantly originates from adipose tissue and has been reported to participate in the OA inflammatory response, although the exact mechanism remains unclear [63]. Past studies have reported that chondrocytes express leptin receptors on the cell surface and that receptor expression is upregulated with increased severity of OA. Leptin may also recruit pro-inflammatory cytokines and collagen-degrading biomarkers, such as IL-1 and MMPs, to the joint space, exacerbating cartilage damage mechanisms in OA [64,65]. A recent genetic study reported a significant relationship between the leptin gene and its receptor gene polymorphisms and OA development in both normal-weight and overweight patients [66]. Additional research is required to elucidate the precise signaling pathway and the clinical utility of leptin in OA pathogenesis. 

### 6.5. Hyaluronic Acid 

Hyaluronic acid (HA) is a non-sulfated glycosaminoglycan ubiquitous in connective soft tissues, such as articular cartilage. HA is synthesized by several connective tissue cells, including fibroblasts, and has been reported to enhance native chondrocyte HA synthesis [62,67,68]. Exogenous viscosupplementation of HA has been established as a beneficial therapy for the alleviation of OA structural damage and clinical symptoms due to its anti-inflammatory and synthetic properties [68]. Compared to healthy joints, lower concentrations of HA are reported in arthritic joints. In addition, exogenous HA has been reported to decrease the production of MMPs and inflammatory cytokines and may also possess anti-nociceptive qualities [69]. Therefore, supplementation with exogenous HA may alleviate pain and stiffness through the restoration of the viscoelastic properties [70]. In a randomized, multicenter study, the AMELIA trial reported that OA patients who received repeated intra-articular HA injections experienced significantly improved functional and pain outcomes compared to those with placebo at a 40-month follow-up [71]. Long-term exogenous HA therapy has been reported as a clinically effective treatment for the alleviation of OA pain and structural dysfunction [70,71].

In addition to the therapeutic benefits, past studies have identified HA as an early biomarker of OA pathogenesis [70]. Singh et al. reported that elevated HA levels effectively identified early knee OA and that HA levels were predictive of clinical disease severity, characterized by WOMAC scoring [68]. Elevated serum HA levels have also been reported to correlate significantly with the total number of arthritic joints in OA [72]. The measurement of HA levels is promising as a tool for diagnosis and determination of clinical severity in OA.

## 7. Future Directions

In order to serve its ideal purpose, a biomarker must have clinical relevance in describing disease progression and/or response to treatment [23]. This review has described a number of current biomarkers that fulfill this function in the analysis and treatment of OA. A 2018 review of OA biomarkers by Watt et al. theorizes that future biomarkers may even be used to mark the start and endpoints of the OA disease process; however, the authors also argue that a biomarker or set of biomarkers have yet to be described that adequately fulfill this function [23]. Therefore, along with continuing to study the many biomarkers described in this review, continuous exploration with novel techniques, e.g., proteomics, metabolomics, etc., is crucial for discovering new OA biomarkers that could potentially achieve the ideal function of outlining the disease process from start to finish [4,23].

Thudium et al. reviewed the way that the study of proteomics has been used in an attempt to explore novel biomarkers for neuropathic pain and how this process could be analogous to a process that similarly employs proteomics to explore novel biomarkers for OA [4]. The only studies that exist to date using proteomics to explore biomarkers have been discovery-oriented, with the aim of looking broadly at protein expression during the disease process of neuropathic pain [4]. However, their review provides the caveat that these proteomic studies do little to elucidate the actual effectiveness of individual proteinaceous biomarkers in describing the neuropathic pain disease process in terms of disease onset, progression, and response to treatment [4]. Furthermore, the same study states that no analogous studies exist to explore OA biomarkers using proteomics; rather, this review merely proposes proteomics as a possible future direction for the exploration and study of OA biomarkers [4].

One overarching theme of the study of biomarkers in describing the OA disease process is the gradual discovery that OA is not merely a localized mechanical process confined to the area(s) of gross chondral decay. Rather, the study of biomarkers has expanded to include the study of inflammatory cytokines and even further to include nucleic acid and protein expression [73]. A review study by Rockel et al. eloquently expands the window of discussion and exploration in OA biomarkers to include the study of local (i.e., synovial fluid sourced) and systemic (i.e., serum sourced) metabolites [73]. The studies that explore local and systemic metabolites to serve as biomarkers for OA encompass a promising area of biomarker study referred to in the literature as metabolomics. While studies have gone as far as to promote certain metabolites as markers capable of describing disease onset, progression, and response to therapy, Rockel et al. demonstrate that these markers are most effective when applied to a subset of OA patients [73]. In essence, focusing on biomarkers that are tailored to individual patient populations (described in this study as OA “phenotypes”) makes many of the currently studied metabolic biomarkers more effective compared to when broadened out to the general public [73].

Despite the significant interest from researchers in recent years, progress in the usage of biomarkers for clinical evaluation and therapeutic development has been slow. Multiple reasons may account for this. First, among the international community, there exists no consensus on the role of biomarkers as an effective surrogate measure for OA, as clinical endpoints have not been uniformly defined [3]. The resulting heterogeneity in the literature poses a significant barrier to the process of biomarker validation. International standardization of biomarkers as a surrogate measure of disease is recommended to accelerate the process from biomarker discovery to clinical implementation [2]. Another challenge is that OA remains a very complex disease with significant variability in the clinical presentation, rate of symptom development, and extent of synovial tissue damage. Clinical trials are also complicated by the fact that many biomarkers under investigation are affected by concomitant disease processes and, therefore, may not have suitable specificity as an OA biomarker. Despite the significant hurdles to the widespread clinical implementation of biomarkers for OA evaluation and therapeutics, there exist significant opportunities for future studies to optimize the evaluation of biomarkers with standardized techniques that allow for the safe and efficient development of new OA treatments.

## 8. Conclusions

Osteoarthritis is an extremely prevalent disease worldwide, and despite advancements in modern medicine, the available treatments for this disease remain almost strictly palliative. The exploration of novel biomarkers for describing OA in terms of its onset, progression, and response to therapy represents a promising and expanding area in the study of OA. The proper discussion of the OA disease process involves a thorough understanding of the pathophysiologic mechanisms behind the progression of OA, especially as it pertains to mechanical damage to the extracellular membrane and the role of matrix metalloproteins in exacerbating local tissue damage. This allows for a discussion of the key molecular mediators in this process. Molecular markers specific to the processes of cartilage degradation, synovial inflammation, and joint injury and inflammation have begun to break the plane between association with a disease process to becoming promising markers for tracking disease progression and response to therapy. Future methods for discovering and analyzing biomarkers to describe the disease process of OA may include proteomics and metabolomics as additional routes for the development of this field.

## Figures and Tables

**Table 1:** Osteoarthritis Biomarkers of Cartilage Degradation and Synovial Inflammation.

	Biomarker	Clinical Relevance
Biomarkers of cartilage degradation in osteoarthritis	Cartilage Oligomeric Matrix Protein (COMP)	Studies have correlated COMP with cartilage degradation that may result in increased severity of OA
	Col2-1 and Col2-1 NO2	Nonspecific biomarker that has been associated with articular cartilage demolition
	C-terminal cross-linked telopeptide of type II collagen (CTX-II)	Urine CTX-II levels have been associated with current joint damage and increased risk of rapid OA progression
	CXCL12 and CXCR2	Elevated CXCL12 synovial levels have been correlated with poor radiographic outcomes in OA patients
Biomarkers of synovial inflammation	Matrix metalloproteinases (MMPs)	Regulates enzymatic cartilage extracellular matrix degradation and increased expression has been reported in patients with OA compared to normal adult cartilage
	Tissue inhibitor of metalloproteinases (TIMPs)	Suppresses activity of TIMPs and represents a promising therapy for OA

Biomarkers that have been established in the pathogenesis of cartilage degradation and synovial inflammation. OA, osteoarthritis.

**Table 2:** Biomarkers of Joint Injury and Inflammation.

Biomarker	Clinical Relevance
Chemokine ligand 2 (CCL2)	CCL2 has been associated with radiographic OA development and may be partially responsible for onset of pain in knee OA
C reactive protein (CRP)	Nonspecific acute phase reactant that facilitates synovial inflammation and may exacerbate clinical OA symptoms
Ghrelin	Neuropeptide that may attenuate the synovial inflammatory response and exhibit anti-nociceptive properties in OA. Elevated ghrelin levels been associated with improved clinical outcomes in OA
Leptin	Studies have reported that Leptin may facilitate the inflammatory response in OA through recruitment of MMPs to the joint space, leading to increased severity of OA
Hyaluronic acid (HA)	Elevated HA levels have been identified as an early biomarker of OA pathogenesis and may be correlated with the total number of arthritic joints in OA

Biomarkers that have been implicated in the inflammatory response of OA. OA, osteoarthritis.

## Data Availability

Not applicable.
